# 

**Published:** 1910-10-08

**Authors:** 


					Just Issued. Ninth Edition, Rewritten and Enlarged, 9s. net.
PHARMACY, MATERIA MEDICA & THERAPEUTICS.
Being a Commentary on the B.P. and its Drugs, with their Preparations,
and on all the new Non-official Remedies in use, with Chapters on the
different processes used in Dispensing, Prescription-writing, Latin
Glossary, &c. By Sir W. WHITLA, M.D., LL.D., Professor, Materia
Medica, Queen's University, Belfast, and Senior Physician, Royal Victoria
Hospital.
By the same Author. New Work. 1,900 pages. Crown 8vo. 25s. net.
PRACTICE OF MEDICINE.
Containing in two Handy Volumes a complete and independent Article on
the Etiology, Symptoms, and Diagnosis of every Medical Disease; arranged
iu Dictionary Form for Practitioners and Students.
London: Baillikise, Tindall & Cox, 8 Henrietta Street, "W.C.
IMPORTANT TO HOSPITAL SECRETARIES.
ANALYSIS LEDGER.
Specially Ruled in accordance with, and adapted to the requirements of
the New Index of Classification recently adopted by the King Edward's
Hospital Fund for London, and which came into operation last year.
The Ledger is bound in half-basil, green cloth sides, printed and ruled in
best style on good paper, and is in every way a serviceable book for Hospital
Price ?1 12s. 6d. Carriage extra.
Secretaries requiring a copy of this Ledger should
order at once to avoid delay.
THE SCIENTIFIC PRESS, LTD.,
28 & 29 Southampton St., Strand, London, W.O.
THE POCKET-BOOK OF TEMPERATURE CHARTS.
Containing Seventeen 12-hour and eight 4-hour Charts, perforated so that eaeh Chart may be easily removed after use.
Bound in paper cover. Suitable size for the pocket. Price 6d. net; by post 7d.
Of all Booksellers, or of
THE SCIENTIFIC PRESS, LIMITED, 28 & 29 Southampton Street, Strand, London, W.O.
BOOKS RECEIVED.
Rebmans.
" Hints for the General Practitioner in Rhinology ancj
Laryngology." By Dr. Johnson Fein.
Swan Sonnenschein.
" Spiritism and Insanity." By Dr. Marcel Voillet.
H. Kimpton. ?
" Care of Children." By B. Myers.
J. Murray.
" Prevention of Malaria." By Ross. > j
" Diseases of the Skin." By Gaucher.
" Philosophies." By R. Rose. i?
University Correspondence College. ,
" London University Guide, 1911."
Hodder and Stoughion.
" Is That Lamp Going Out ?. "
Obituary.
The death is announced of Dr. Joseph Higham HilL
He studied at Edinburgh, taking his L.E.C.P. in 1867,
and the L.F.P.S. at Glasgow in the same year. In 1868
he became an L.S.A., and four years later was admitted
a fellow of the R.C.S. of Edinburgh. His work called
him abroad, and at the University of Brussels he gradu-
ated M.D. in 1879.
Mr. John Aylward Churchill has died at Swatow, ira
China, of hydrophobia. He was only thirty-five years of
age, and took his diploma in 1900.
The seventy-first anniversary festival in aid of the funde
of the Newsvendors' Institution will be held at De Keyser's
Royal Hotel, in the City of London, on " Hallowe'en'T
Monday, October 31. Lord FabeT (Chairman of the York-
shire Post) has kindly consented to preside; his Lordship
not only pleads for charitable consideration of the stricken
workers in this laborious trade, but seeks to emphasise tha
national scope of their Society's beneficent operations.
The Progress of Scientific Discovery in psychology-
is a fascinating study, and in "TI10 Evolution of Mind,"
which is shortly to be issued by Messrs. A. and C. Black,
Mr. Joseph McCabe shows how much light has been thrown
on this department by recent discoveries. With a fresh
outlook the most recent conclusions are challenged in regard
to mind in the lower animals. Mr. McCabe has paid
special attention to the earliest dawn of mind and the
momentous influence of geological changes on the appear-
ance of higher types of mind, and the passage from non-
human to human intelligence.
The Subject of Heredity is interesting to all thinking
men. Messrs. A. and C. Black announce a new book on the
subject entitled " First Principles of Heredity," by Dr. S.
Herbert. Although th'e subject is treated in a thoroughly
scientific fashion the book has been written for the average
educated reader, and assumes no previous knowledge of the
subject on the reader's part, and deals with the following
subjects : The Need of the Science of Heredity, Reversion,
Determination of Sex, etc.; The Inheritance of Acquired
Characters; The Inheritance of Disease; Mendelism,
Biometrics, and Heredity versus Environment (Eugenics).
The book will be well illustrated.
The Historical Section of Sir Walter Besant's great
survey of London, which has been issued in seven volumes,
ranging from "Early London" to "London in the Nine-
teenth Century," is now to be followed by the topographical
section beginning with a volume entitled " The City," which
Messrs. A. and C. Black announce among their forthcoming
books. The greater part of this new work is written by Sir
Walter Besant in his delightful style born of the enthusiasm
he felt for the survivals of the various Londons which have
stood on the pieces of land known as the City. Sir
Walter was never happier than when wandering through
the City's by-ways in search of traces of seventeenth-century
and earlier buildings which in spite of the fire and constant
reconstruction were unexpectedly found a few years ago.
The vast amount of knowledge patiently acquired through
years of labour has, in this new volume on the City, been
carefully put together, and now forms a most attractive and
permanently valuable description of London past, passing,
and present. The illustrations will be numerous, and there
will also be reproductions of old and new maps and plans of
various parts of the City.
The Right Rev. the Lord Bishop of Oxford will dis-
tribute the prizes to students of the Royal Dental Hospital
of London on Wednesday, October 12, 1910, at 8 r.M.
precisely, at the Royal Institute Galleries, Princes Hall,
Piccadilly.
October 8, 1910. THE HOSPITAL 61'
Gifts and Bequests.
The treasurers of the Middlesex Hospital have received
an anonymous donation of ?120 to the Cancer Ward Fund.
Prince Francis of Teck has received the following on
ibehalf of the Middlesex Hospital: Sir George Cooper,
?200; Mr. Edward E. Sassoon, ?50; Mr. William McEwan,
?50.
The late Mr. Edward Matyear, of the Crabtree Farm,
Fulham Palace Road, market gardener, has left the residue
of his freehold estate, more than twenty-one acres, with a
river frontage of 500 ft., valued at ?4,000 an acre, to King
Edward's Hospital Fund. The legacies, free from any
?duty, include ?500 to the West London Hospital.
Mr. William Henry Kirkham, of Keymer, Sussex, who
ieft estate of the gross value of ?35,225, has bequeathed
?200 each to the Sussex County Hospital and the Eoyal
Alexandra Hospital for Children, Brighton; ?100 each to
the Brighton, Preston, and Hove Dispensary, the Brighton
Throat and Ear Hospital, and the Hospital for Women,
Brighton.
Mr. William Bell, Bootle, Liverpool, a team owner, who
Jeft estate valued at ?165,635 4s. 8d. gross, has bequeathed
?1,700 to each of his two female servants ; ?1,000 each to the
Bootle Borough Hospital, the David Lewis Northern Hos-
pital, Liverpool, the Royal Southern Hospital, Liverpool,
the Stanley Hospital, Kirkdale, and to the Liverpool Royal
Infirmary.
Lady Margaret Anne Harriet Moore, of Hyde Park
Gate, Kensington Gore, S.W., who died on August 28,
widow of Surgeon-General Sir William James Moore, who
left estate valued at ?70,954 gross, has bequeathed ?100
each to the Children's Convalescent Horn?, Glynde,
Sussex; the Children's Convalescent Home, East South-
sea; the Children's Convalescent Home, Great Yarmouth;
the North of England Sanatorium for Children, South-
port ; the Craig Convalescent Home for Children, More-
cambe; the Convalescent Home for Poor Children, St.
Lecnards-on-Sea; and to St. Mary's Convalescent Home,
Eirchington-on-Sea.
Jottings.
The Duke of Westminster is lending Grosvenor House
for a drawing-room meeting to discuss a scheme for a
memorial to Miss Nightingale.
The new Federal Laboratory (Melbourne), where tea-
testing and all Customs analyses are to be conducted, is on
the point of completion. The building has cost ?4,542,
and furniture and fittings ?863.
A successful parade and carnival, entirely organised
by working men, was held in Gravesend last Saturday
in aid of the hospital, which will benefit to the extent
??f nearly ?100 as a result of the same. The prizes in con-
nection with the carnival were presented to the successful
competitors by Sir Gilbert Parker, M.P., in the Borough
Market Hall on Wednesday, when the band of the 1st Duke
of Cornwall's Light Infantry.gave a concert..
At a conference held at Shrewsbury, representative of the
whole of Wales, and presided over by the Lord Mayor of
Cardiff (Alderman Chappell), to consider a Welsh national
memorial to the late King, Mr. David Davies, M.P., who
Shas urged a scheme to fight consumption and has offered
?30,000, said that he had promises of ?140,000, but the
cum required would be ?300,000. The meeting approved
of the principle of a national campaign to stamp out
consumption.
A gift of scientific apparatus for use in the investigation
of nervous diseases has been made to the National Hospital
for Paralysis and Epilepsy, Queen's Square, by the rela-
tives and friends of the late Dr. E. C. Beevor, F.R.C.P.,
who for many years was connected with the hospital.
Complaints regarding the business relations of charitable
institutions with contractors who are members of the com-
mittees of such institutions having been investigated and
found to have some foundation, a clause dealing with the
question is to be inserted in the Charities Bill which the
Victorian State Treasurer proposes to introduce.
The Birmingham Daily Mail King Edward Memorial
Fund, which was opened four months ago, was closed last
Wednesday. ?27,516 7s. 7^d. has been raised, of which
it is proposed to spend ?2,000 upon a statue of King
Edward to be erected in Victoria Square, and the remainder
will go towards the erection of a new memorial hospital
for children.
A "Million Penny Fund" has been adopted at West
Bromwich District Hospital to wipe off the heavy debt
(amounting to nearly ?3,000) on the hospital, and also
to endow a bed in that institution in memory of his
late Majesty King Edward VII. The Mayors of West
Bromwich and Wednesbury and the Chairman of the Old-
bury District Council are starting subscription lists in their
respective districts.
On the proposal of the Mayor, Councillor Walter Gaskell,
the Hornsey Town Council has decided to commend to the
burgesses, by way of memorial to King Edward, the col-
lection of money to enlarge the site of the Hornsey Cottage
Hospital and secure the freehold interest, and for pro-
viding an endowment. The hospital was opened last
January in commemoration of King Edward's Corona-
tion.
In a recent lecture Mr. R. C. Norman, secretary and
superintendent of the Alfred Hospital, Australia, referred
to a scheme for hospital maintenance which he had out-
lined some years ago : Those earning 10s. a week and
under to be exempt; 10s. to 20s. to pay one halfpenny a
week; and so on up to 50s. to 60s.?3d. a week. For
incomes of over ?3 a week there should be a specially
earmarked income tax of Id. in the ?.
Under the presidency of Princess Louise the postponed
pageant of London will be produced by Mr. Frank Lascelles,
for which 15,000 citizens of London and delegates from
all the Colonies have been enlisted. There will be an
Imperial Exhibition, in which the Governments of the
overseas Dominions will co-operate, and also an imperial
sports meeting. The proceeds of the undertaking will be
devoted to the King Edward VII. Hospital Fund.
A meeting of citizens of Grimsby has been held, the
Mayor (Councillor Roberts) presiding, at which a resolu-
tion, proposed by the High Steward of the borough (Lord
Heneage), supported, by the Earl of Yarborough and Mr.
G. S. Letten, was carried unanimously that a local King
Edward VII. Memorial Convalescent Fund" should be
raised to aid convalescent patients from the Grimsby and
District Hospital to recuperate at convalescent homes. It
is hoped to raise at least ?2,000.
TO HOSPITAL SECRETARIES AND OTHERS.
We invite contributions from any of our readers, and
shall be glad to pay, according to length, for items of news
for the institutional section (including appointments, resig-
nations, gifts, etc.) which we may publish. All payments
for manuscripts used are made as early as possible after the
beginning of each quarter.
THE FUTURE ?. HOSPITALS
MEDICAL RELIEF FOR ALL ACCORDING TO MEANS.
A Scheme of Co-operation between the PATIENTS, the HOSPITALS,
and the STATE.
Price 2d. By Sip HENRY BURDETT, K.C.B., K.C.V.O. Price 2d.
THE SCIENTIFIC PRESS, Ltd., 28 & 29 Southampton St., Strand, London, W.C.,
and at all Bookstalls.
GENERAL PRACTITIONERS'
CONTRIBUTIONS.
The Hospital devotes a special page to General
Practitioners' contributions. We therefore invite
from practitioners contributions based upon their
experience in the management of cases, and in the
treatment and diagnosis of disease; especially shall
we be prepared to welcome articles dealing, practi-
cally, with treatment, and with the use and value
of new remedies and methods.
No article should exceed 1,100 words, and, if
accepted, one guinea for a contribution of this
length will be paid to the writer after publication.
Each communication should be accompanied by a
stamped directed envelope for the return of the MS.
if found unsuitable.
Contributions should be written, or preferably
typed, on one side of the paper only, and all articles
sent in are accepted upon the distinct understanding
that they are forwarded to The Hospital only.
BUSINESS NOTICES.
Annual Subscriptions.
The rate of annual subscriptions to The
Hospital, either direct from the Office or through
agents, is: ?
For the United Kingdom  15s. a year.
For the Colonies and Abroad ... 17s. ,,
For the United Kingdom (with
Insurance Policy)  ?1 >>
inclusive of postage in each case.
Letters relating to the Publishing, Sale and
Advertisement Departments must be addressed to
the Manager (not to the Editor): ?
The Manager,
The Hospital Building,
28 and 29 Southampton Street,
Strand, London, W.C.
THE SCIENTIFIC PRESS PUBLICATIONS
MEDICAL HISTORY FROM THE
EARLIEST TIMES.
By E. T. Withington, M.A., M.B. Oxon. Demy 8vo.
over 400 pages, with 2 Plates, cloth gilt, 12s. 6d. net;
12s. lOd. post free.
PHOTOMICROGRAPHY.
By Edmund J. Spitta, L.R.C.P. Lond.; M.R.C.S. Eng.,
F.R.A.S. Demy 4to. with 41 Half-tone Reproductions
from Original Negatives and 63 Illustrations in the Text,
cloth gilt, 12s.
THE SCHOTT TREATMENT FOR
CHRONIC HEART DISEASES.
By Richabd Greene, F.R.C.P. Ed. Second Edition,
\ Revised, demy 8vo. paper cover, 6d.
THE CONSUMPTIVE WORKING MAN
What can Sanatoria do for him ?
By Noel Dean Bauds-well, M.D., Medical Superintendent,
King Edward VII. Sanatorium, Midhurst. Demy 8vo.
illustrated, cloth, 10s. 6d. net; 10s. lOd. post free.
SANATORIUM TREATMENT OF
CONSUMPTION.
By T. N. Kelynack, M.D., M.R.C.P. Crown 8vo. limp
cloth cover, 6d. net; 7d. post free.
THE SELECTION OF CONSUMPTIVE
CASES for SANATORIUM TREATMENT.
By T. N. Kelynack, M.D., M.R.C.P. Crown 8vo. limp
cloth cover, 6d. net; 7d. post free.
28/29 SOUTHAMPTON STREET, STRAND, LONDON, W.C.
THE IDEAL WORK OF REFERENCE.
BURDETT'S HOSPITALS & CHARITIES
1910 EDITION.
?be U?eai%46ook of philanthropy anfc Ibospital annual.
Edited by SIR HENRY BURDETT, K.C.B., K.C.V.O.
A Complete Guide and Directory of the Hospitals, Asylums, and Charitable Institutions
throughout the English-speaking World.
Contains Special New Chapters on :?
Voluntary Hospitals in 1909.?Soundness of the Voluntary System.?The need of greater zeal on the
part of those who accept office.?State-aided Hospitals in the U.S.A. and Canada.?The Appropriation
?of Public Funds for the partial Support of Voluntary Hospitals in U.S.A. and Canada.?The Need of an
Apostle and of greater interest on the part of the Clergy, &c., &c.
TWENTY-FIRST' YEAR OF PUBLICATION.
Over 1,000 pages. Price 10/6 net; Post Free, 10/11.
ORDER youp copy of the Medical Whittaker NOW,
Publishers:
THE SCIENTIFIC PRESS, LIMITED, 28 & 29 Southampton Street, Strand, London, W.C.

				

